# Student midwives’ perceptions on the organisation of maternity care and alternative maternity care models in the Netherlands - a qualitative study

**DOI:** 10.1186/s12884-016-1185-4

**Published:** 2017-01-11

**Authors:** J. Catja Warmelink, T. Paul de Cock, Yvonne Combee, Marloes Rongen, Therese A. Wiegers, Eileen K. Hutton

**Affiliations:** 1 Department of Midwifery Science, AVAG and the EMGO Institute for Health and Care Research, VU University Medical Centre, Amsterdam, The Netherlands; 2 Midwifery Academy Amsterdam Groningen, Amsterdam/Groningen, The Netherlands; 3 The Bamford Centre for Mental Health and Wellbeing, University of Ulster, Coleraine, UK; 4 Netherlands institute for health services research (NIVEL), Utrecht, The Netherlands; 5 Faculty of Health Sciences, McMaster University, Hamilton, Canada

**Keywords:** Integrated care, Maternity care, Midwifery, Primary care, Qualitative research

## Abstract

**Background:**

A major change in the organisation of maternity care in the Netherlands is under consideration, going from an echelon system where midwives provide primary care in the community and refer to obstetricians for secondary and tertiary care, to a more integrated maternity care system involving midwives and obstetricians at all care levels. Student midwives are the future maternity care providers and they may be entering into a changing maternity care system, so inclusion of their views in the discussion is relevant. This study aimed to explore student midwives’ perceptions on the current organisation of maternity care and alternative maternity care models, including integrated care.

**Methods:**

This qualitative study was based on the interpretivist/constructivist paradigm, using a grounded theory design. Interviews and focus groups with 18 female final year student midwives of the Midwifery Academy Amsterdam Groningen (AVAG) were held on the basis of a topic list, then later transcribed, coded and analysed.

**Results:**

Students felt that inevitably there will be a change in the organisation of maternity care, and they were open to change. Participants indicated that good collaboration between professions, including a shared system of maternity notes and guidelines, and mutual trust and respect were important aspects of any alternative model. The students indicated that client-centered care and the safeguarding of the physiological, normalcy approach to pregnancy and birth should be maintained in any alternative model. Students expressed worries that the role of midwives in intrapartum care could become redundant, and thus they are motivated to take on new roles and competencies, so they can ensure their own role in intrapartum care.

**Conclusions:**

Final year student midwives recognise that change in the organisation of maternity care is inevitable and have an open attitude towards changes if they include good collaboration, client-centred care and safeguards for normal physiological birth.

The graduating midwives are motivated to undertake an expanded intrapartum skill set. It can be important to involve students’ views in the discussion, because they are the future maternity care providers.

## Background

The Dutch maternity care model has been held out as example of how to slow or reverse the march towards medicalisation of birth and technology driven specialist midwifery and maternity care [1–5]. However, unexpected high perinatal mortality reported at the turn of the 21st century raised concerns about the quality of the Dutch maternity care system resulting in a call for system change to enhance care [6–10]. Organisation, inter-professional relations and coordination have been identified as factors which may disrupt the smooth functioning of the maternity care. Issues such as a lack of a shared maternity notes system, misaligned financial incentives, different perspectives on antenatal health and suboptimal inter-professional communication have all been identified as contributing to systemic disorganisation [6]. Recent research indicated that clarity on each profession’s role and responsibilities within the collaboration seemed to be lacking and that many professionals did not perceive themselves as being an integral part of a team [7]. There has been a considerable rise in non-urgent referrals during labour from primary midwife-led care to obstetrician-led care [8, 9], challenging the sustainability of the current echelon system in the Netherlands with its strict role division between primary and secondary maternity care [6–10]. Therefore, major changes in the organisation of maternity care in the Netherlands are being considered, moving from an echelon system where midwives provide primary care in the community and refer to obstetricians for secondary and tertiary care, to a more integrated maternity care system involving midwives and obstetricians at all care levels [5, 11].

### Current organisation of maternity care in the Netherlands

Like all health care, maternity care in the Netherlands is organised in echelons, with a strict role division between primary and secondary/tertiary care. The independent primary care midwife plays a key role as provider of standard maternity care in the Netherlands and provides one-to-one care to women during pregnancy, birth and the postpartum period in individual or group practices of midwives [12]. Primary care midwives have a gate keeping role: in the event of complications, an increased risk of complications, or a request for pharmacological pain relief, midwives transfer care of women to secondary care in a general hospital or to tertiary care in an academic referral centre, both with obstetricians and clinical midwives, who work under the responsibility of obstetricians. The Obstetrics Indications List [13] distinguishes between ‘physiological’ and ‘pathological’ pregnancies and births and directs all such referrals. There are tasks and responsibilities that currently fall outside the scope of primary care midwifery in the Netherlands, such as supervising medium risk pregnancies (obese or diabetic clients, clients with thyroid problems) and medium risk births (meconium-stained liquor, previous Caesarean section, pharmacological pain relief), and skills as monitoring the fetus with cardiotocography (CTG). Healthcare insurance companies reimburse secondary or tertiary care exclusively after referral for medical reasons [14]. In 2013, 85.4% of all pregnant women in the Netherlands started antenatal care with a primary care midwife, 50.6% started labour with a primary care midwife and 28.6% of all births (*n* = 167,159) were supervised by a primary care midwife at home or in a hospital or birth centre [15]. The Netherlands has more midwives (n = 2692) than specialists in obstetrics and gynaecology (n = 882) [16, 17].

### Call for other forms of cooperation

Other countries took the rather unique Dutch maternity care system as an example for changing their maternity care systems [1, 9]. In these countries, such as New Zealand, Canada and the United Kingdom, midwives increasingly work autonomously and the homebirth rate is rising. Conversely, the quality of care of the ‘Dutch’ way of organising maternity care has been more and more questioned. Following reports of higher than anticipated perinatal mortality in the Netherlands [18], a government appointed Steering Committee [19] released its advisory report called ‘A good start’. Based on stakeholders’ opinions this committee presented a set of recommendations on the direction in which the Dutch maternity care should evolve in order to halve the perinatal mortality figures. The report contained the directive to improve the quality of maternity and perinatal care by encouraging closer cooperation and better communication between all maternity care professionals. Other recommendations by the Committee included local execution of multidisciplinary protocols developed on a national level and prevention of caregiver delay, specific attention for disadvantaged women, shared decision making and the accessibility of 24/7 maternity care.

Improved collaboration can be realised by implementing alternative maternity care models, such as integrated care in a joint venture with midwives and obstetricians (vertical integration), shared care within primary care (horizontal integration), or midwives working in the community as well as in the hospital [10, 20–22]. Health insurance companies supported this approach by strongly advising midwives and obstetricians to collaborate in a professional as well as a financial partnership [10]. Other forms of cooperation are feasible and a variety of maternity care models are being tested as pilots for a national model [7, 23]. There is, however, currently limited evidence to support any of the pilot approaches and no consensus among professionals and other stakeholders about whether, how and when the maternity care system should be integrated [7, 23–26]. Furthermore, the concept of integrated care is ambiguous and often used as an umbrella term [27]. Different stakeholders have given their opinions on the matter of integrated care [10, 11, 25, 26]. Although student midwives have been exposed to these different models in recent years, their voices have not been included in the conversation about change. Student midwives will be entering a changing maternity care system and it is important to involve students’ views in the discussion and the transitions, because they are the future maternity care providers with a unique perspective as semi-outsiders in the system who may have innovative perspectives as newly integrated members of the system. Further, it is important to gaining buy-in for any proposed change from future midwives. Final year students will have been part of various situations in their internships in different places in primary, secondary and tertiary care and were in that way in a position where they can observe what works and what does not work in the field. To our knowledge, no research has been conducted to investigate the views of midwifery students with regard to the reorganisation of maternity care.

### Aim of the study

The purpose of this research was to explore the perceptions of graduating students regarding the organisation of maternity care and alternative maternity care models in the Netherlands. These student midwives have studied for 4 years, with 2 years in internships in primary, secondary and tertiary care and have witnessed new developments and have become part of the change. Findings from our study add the perspective of an important group of future professionals on how they see themselves fit in a changing system, which can further inform the direction of the current policy dialogue on the development of maternity care in the Netherlands. Our research question was: What are the perceptions of final year midwifery students in Amsterdam (VAA) and Groningen (VAG) on possible future forms of cooperation in maternity care, including integrated care?

## Methods

### Design

This qualitative descriptive study is based on an interpretivist/constructivist paradigm using a grounded theory design [28, 29]. This was an appropriate approach as our research question has a broad scope and there is relatively little prior knowledge about perspectives in the group under investigation. We conducted both individual interviews and focus group interviews with student midwives, letting these two methods complement each other [29]. Particularly delicate topics were more readily discussed in individual interviews covering maximum depth; whereas in a group interview, a more dynamic interaction between participants generated new ideas or evoked new insights that might not have been thought of in a one to one interview.

### Recruitment and sample

Eligible participants were 79 final year student midwives of the Midwifery Academy Amsterdam Groningen (AVAG). From these female students, 45 studied in Amsterdam (VAA) and 34 in Groningen (VAG). Students were recruited from the AVAG on a web-based learning system used by schools for giving instructions to students and by approaching them face-to-face. To achieve variation in our sample, final recruitment was targeted at specific categories of student midwives, such as those who did not have an intention to work as active practising midwife, or had a child of their own. In total, 18 students participated; ten in individual interviews (seven in Amsterdam; three in Groningen) and eight (three and five respectively) students in two focus groups (Groningen). We intended to interview until we reached saturation of concepts.

### Data-collection

Information about the research project and interview procedure was provided on a web-based learning system and again face-to-face before the interview or focus group meeting. See our ethical/consent statement (Declaration B) for details on ethics approval and consent to participate. A topic guide (Table 1) was used to frame the questioning during the interviews to ensure key areas were discussed. If necessary, further exploratory questions were asked (for example, on the behavioural determinants: knowledge, norms, attitudes, and intention [30] and experiences). Six semi-structured interviews with midwifery students in Amsterdam (VAA) were conducted in the spring of 2014 by student midwives (MN and YC) after a 5-days training on interviewing and qualitative research and under supervision of the lead author, a psychologist and experienced interviewer (CW). Four interviews and two focus groups at the Midwifery Academy Groningen (VAG) were conducted by CW in the summer and autumn of 2014.

**Table 1:** Topic guide

• How can midwifery be ‘future proof’ in the eyes of student midwives? • How do student midwives see the role of midwives and their future role in the (current) maternity care model? • What are their hopes and fears with regards to alternative maternity care systems?	

To increase validity of our findings, we used different source materials (data-triangulation), diverse researchers (investigator-triangulation) and a varied group of participants (negative case analysis). The participants were encouraged to speak freely about their experiences. The interviewers stressed their neutrality by exploring both positive and negative remarks of the participants. The interviews lasted 35 min on average (range: 18–56). At the conclusion of each interview the participants were invited to provide feedback on the interview and to verify a short oral summary. Directly after each interview the interviewers evaluated their findings and formulated areas that called for more in-depth exploration in the next interview. We kept a logbook (audit trail) in which we indicated with whom we had a conversation and about what or whether there were any special circumstances, where the conversation took place and what was going on in the media.

Participating students were individually invited by email to comment on the completed transcripts and preliminary results (member checking). All participants read, checked and approved these transcripts and results. Describing the results criteria [31] for reporting qualitative research (COREQ-criteria) were used. The final subthemes and main themes were discussed in a group session of health care professionals at conferences and with midwifery lecturers (peer debriefing).

### Data analysis

Descriptive analyses were used to describe characteristics of the study population. The interviews were recorded on tape, transcribed and anonymised. The analyses of the qualitative data were done in a successive and cyclical order, using constant comparison/grounded theory design [28, 31]. The first six transcripts were analysed independently by all three interviewers, then reviewed to reach consensus on interpretation of the findings and to reflect on the research process and the role of the researchers. The transcripts were read several times to get a sense of the content of the whole. The material was first organised into information units and open coded (labelling), then axial coded (categorized) and finally selectively coded (thematically). More than one label or category could be added to a fragment of text. This analysis of the first six transcripts led to new ideas, which were used when again conducting new interviews with a number of students. These rounds of analyses were continued until we felt no new knowledge had or would present itself. We described the themes, compared the differences, recorded meaningful associations and compared the findings with existing literature. Examples of the analytical coding process are shown in Table 2. The analysis indicated data saturation, which means that the inclusion of further data would probably not have resulted in the identification of new themes.

**Table 2:** Examples of coding process

1^st^ level Fragment	2^nd^ level Labelling	3^rd^ level Category	4^th^ level Theme
*“We [students] aren’t tied to anything, I mean, we can come and go where we want, we don’t have any fixed… I think we’re much more flexible there, that we’re less concerned about precisely how things will be done, as compared to someone who has been running their own practice for twenty years and has established themselves in the field like this.” #fg2*	Not stuck Flexible	Open to change	Students are open to change
*“The media… that so much negative emphasis is placed on home births and that pregnant women now even find it more attractive to just (go to the hospital) #7*	Bad media coverage Shift toward medicalisation	Society: Pressure to change	Students feel change is inevitable
*“…a healthcare system that centres on the client, but then a self-determining client who also has confidence in herself again, and I think that the primary midwife has a great deal of expertise for restoring this confidence.” #4*	Client centered Empowerment	Essential components	Students accept change with conditions

Representative quotations were chosen to demonstrate the themes and subthemes identified; these are presented in the RESULTS section, each followed by the participant or focus group number. The quotes were translated into English by an accredited translator, and then translated back in Dutch by another investigator in order to check for validity of translation.

## Results

In total, 18 students participated. The participants were between 21 and 28 years of age (mean = 23.4 years), one student was a mother. One student wanted to work as a researcher after graduation; the others all wanted to work as primary care midwives. All but one started the midwifery training in August 2010, one student started in August 2009.

The data from the interviews identified four key issues in student midwives’ perception of the organisation of maternity care.

### Schematic representation of the results

1. Students are open to change
  - Open attitude towards change
    i) Not stuck
    ii) flexible
  - Examples of maternity care models
    i) the current echelon system
    ii) other maternity care models
      1. shared care within primary care (horizontal integration),
      2. shared care between primary and secondary care (vertical integration),
      3. midwives active in the community as well as in the hospital
      4. CenteringPregnancy ^TM^ (group antenatal care).

2) Students feel change is inevitable.
  - Pressure from government and health Insurance companies
  - Decline in numbers of home births,
  - Social trend towards medicalisation
  - Bad media coverage
    i) home birth
    ii) perinatal mortality figures
  - Resentment, lack of trust, power imbalance and ‘professional territorialism’ between occupational groups
    i) obstetricians vs. midwives
    ii) Primary care midwives vs. clinical midwives.

3) Students accept change with conditions
  - Provide the best possible care
  - Essential components
    i) good collaboration, mutual trust and respect
    ii) client-centered care, empowerment
    iii) safeguard of normal birth, (pregnancy and childbirth is a fundamentally physiological process)
    iv) shared maternity notes system
    v) shared guidelines and protocols
    vi) emergency assistance accessible.

4) Students need some reassurance about their role
  - Profession could become redundant
  - Interested in taking on new tasks and learning new skills
    i) supervising medium risk pregnancies
      1. obese or diabetic clients, clients with thyroid problems, etc.
    ii) supervising medium risk births
      1. births with meconium-stained liquor, previous Caesarean section, pharmacological pain relief, etc.
    iii) pick up new skills,
      1. prescribing birth control, doing cardiotocography (CTG), vacuum extraction in primary care, etc.

### Theme 1: Students are open to change

Although primarily trained as primary care midwives, the students had experienced examples of different approaches to care as part of their clinical educational trajectory with both the current echelon system as well as other, more integrated, maternity care models such as shared care within primary care (horizontal integration), shared care between primary and secondary care (vertical integration), midwives active in the community as well as in the hospital.

- *[I envision a] birth centre… a concentration of care, with the maternity care assistant, ultra sound centre, …a whole team of health care providers, not only obstetric care. [I had my internship..].. where the midwives were working in primary care as well as in the hospital. One day as primary care midwife, the next day as clinical midwife. The obstetricians in the hospital …as rear guard to do physiological (!) births. #6*

In principle the students said they were open to change, possible because they were not yet established in their practice habits and had no financial obligations. The following quotes illustrate some of the student ideas about types of change and their openness to system change:

- *Well, I think that a new generation of midwifery students.. approach things in a fresher way and can also play a role in making this more usual but ...... in consultation and amicability, but sure, it will take a few years to overcome the old resentment, I think ” #2*
- *So I think, yes, progress is always good of course… I just wonder whether it’s worth radically changing the whole system right now. #1*
- *Obstetricians need midwives and midwives need obstetricians. So it should then basically … turn out OK. #5*

### Theme 2: Students feel change is inevitable

The students assumed that the way in which maternity care will be organised in the Netherland is about to change. Students identified reasons for the perceived inevitability of change including the pressure from government and health insurance companies, the overall decline in numbers of home births, a social trend towards medicalisation, the negative media coverage about home birth which the media erroneously related to the relatively high perinatal mortality figures, and the resentment, lack of trust, ‘professional territorialism’ and power imbalance between occupational groups (obstetricians versus midwives; primary care midwives versus clinical midwives). Highlights of the key aspects are evident in the following quotes:

- *…especially the insurers, in my view, want things to get better and otherwise they just don’t pay. I think that pressure is coming particularly from the insurers and from the state #2*
- *…healthcare insurers are so incredibly powerful … pretty much omnipotent. #9*
- *…because it has also been a bit of a trend in other countries, where you see that pregnancy and birth are much more medicalised. So I can imagine that the same is going to happen here, too. #1.*
- *I think that people are also much less used to giving birth at home… # 5*
- *Very often I have the feeling that the obstetrician thinks ‘you want to be autonomous, well you can just look after yourself then!’ And if you want to discuss something with this person, …they first want to see the client and once they’ve seen the client, they basically already take over the whole thing. #fg1*
- *..you can clearly see that the secondary healthcare providers are trying to get a bit more power in the field of maternity care, and what you see is that midwives can quickly get pushed aside in the primary field #fg2*

### Theme 3: Students accept change with conditions

Students identified that an important condition to accepting any change in organisation of the maternity care system was, that they could (in their soon to be assumed role as primary care midwives) be able to provide the best possible care. Participants indicated that good collaboration between professions, including a shared maternity notes system and guidelines, and mutual trust and respect, were important aspects of any alternative model. Client-centred care, and the safeguard of the physiological, normalcy approach to pregnancy and birth should be maintained in any alternative model.

- *But sure, if it’s proven that the client could get better care from… that other system, integrated care, then I would … yes … change #3*
- *I also think it’s a good thing if, for instance, obstetricians have consultancy hours in a midwife’s practice, meaning they get out of the hospital. And that midwives can indeed also go into hospitals to handle births with a slightly higher risk. #2*
- *Perinatal mortality in the Netherlands … could also simply be reduced through better communication and better collaboration #8*
- *…of essential importance ..is … midwifery wisdom … that you look holistically at the person herself #9*
- *in other healthcare areas … integrated health care is indeed applied… but the big difference is … if you’re pregnant, then you’re not ill! You’re quite simply healthy and so why should you have to go into hospital? #3*

### Theme 4: Students need some reassurance about their role

Although the students said that they could support change under certain conditions, they feared that their primary care role in midwifery could become redundant or undermined and that the role of midwives could be marginalized. The students were afraid that, for instance, the midwives might only provide prenatal and postpartum care but no intrapartum care in the future.

As an anticipatory response to these concerns, students felt that there might be benefit in expanding their role and competence and to take on new tasks and responsibilities in primary care, some of which are common tasks for midwives in other countries but that currently fall outside the scope of primary care midwifery in the Netherlands. They gave examples such as: supervising medium risk pregnancies (obese or diabetic clients, clients with thyroid problems) and medium risk births (meconium-stained liquor, previous Caesarean section, pharmacological pain relief), and adding new skills, such as prescribing birth control, monitoring the fetus with cardiotocography (CTG) or doing vacuum extraction.

- *I think, how things will turn out, I think that in the end we’ll be allowed to do less. I think that ultimately the midwives will be permitted to do less and less and for instance that births… will be passed fully to what is now secondary healthcare and that we, for instance, will only do the prenatal checks. But that’s pretty much the worst scenario. But it’s possible. #1.*
- *The more skills and actions you give up, then at a certain moment the less remains of your own professional profile. So maybe we should actually make sure we take on more tasks. #fg1*

## Discussion

This study aimed to explore student midwives’ perceptions on the current organisation of maternity care and alternative maternity care systems. Exploring the perspectives of students regarding the evolving role of midwives can help inform policy and enhance conversations about proposed system change. Our exploratory qualitative study adds to the evidence on barriers and facilitators to inter-professional collaboration in maternity care and provides a timely contribution to the literature as Dutch maternity care faces significant health system changes. Student midwife views contribute to a multifaceted understanding of how different echelons of care currently work together and how integrated care would impact women’s childbirth experiences.

Our descriptive thematic findings indicate that students perceived an inevitability regarding change in the organisation of maternity care, going from an echelon system with primary, secondary and tertiary care, to a more integrated maternity care system. Participants pointed out that good collaboration between professions, including a system of shared maternity notes and guidelines, and mutual trust and respect were important aspects of any alternative model. Students indicated that client-centred care, and the physiological, normalcy approach to pregnancy and birth should be safeguarded and maintained in any alternative model. Students worried that the role of midwives in intrapartum care may become minimised, and thus they are motivated to expand their scope of practice in order to enhance their role in providing intrapartum care.

We found that midwifery students had experienced a range of approaches identified in the literature as defining elements of models of maternity care including [32]: who provides the care (obstetricians, midwives, allied care providers), whether the providers are known to the woman, where the care occurs (at home, in hospital, community venue), when the care occurs (gestation at booking, frequency and length of visits, after hours contact), and how the care is provided (one-to-one or group visits) and as described as possible future maternity care models for Dutch practice [23]. Students identified shifts away from the original ‘echelon model’ and indicated openness to such change (theme 1). As in an explorative study among clinicians, working in community practices as well as in academic practices in maternity care in the USA in 2011 [33], when students in our study indicated a preference for a specific maternity care model, the motivation was mainly related to the desire to provide the best possible maternity care.

One of the reasons for the perceived inevitability of the change in organisation of maternity care (theme 2), according to the students, was the resentment, lack of trust and professional territorialism between midwives and obstetricians. Like our participating students, midwives have been found to report a power imbalance in which they feel to be viewed as inferior to obstetricians [9, 10]. A perceived power imbalance could harm inter-professional collaboration and may cause the experienced lack of trust [34]. The negative effect of the perceived power imbalance might be exacerbated by the obstetricians’ reported lack of knowledge about the midwives’ responsibilities and activities [10, 35]. Midwifery students in the Netherlands have some knowledge of the obstetrician’s and clinical midwives’ roles, yet medical students are rarely introduced to the roles of primary care midwives before they are required to work with them.

Our findings of essential components which are conditional for successful collaborative practice (theme 3) - such as the call for good collaboration between maternity care providers in mutual trust and respect; client-centered care and continuity of care; safeguarding of normal birth; a shared maternity notes system and shared guidelines - were also seen in other studies [9, 10]. Students are in a position where they can observe what works and what does not work in the field, because they were part of various situations in their internships in different places in primary, secondary and tertiary care.

Among Dutch maternity care professionals there is a lack of consensus regarding the distribution of responsibilities and tasks (theme 4) for moderate risk indications [7]. From 2000 to 2008, there was a considerable rise in non-urgent referrals from primary midwife-led care to obstetrician-led care during labour [6] and most referrals were for moderate risk indications [36]. Our study highlights that students see a need for expanding a primary care midwife’s responsibilities and competencies, similar to maternity care systems in other countries, such as the United Kingdom and Canada, where the midwife providing care to low risk woman commonly remains the caregiver when certain moderate risks occur [7, 24].

Lowering perinatal mortality was the main driver of changes in maternity care over the past decade in the Netherlands. The value of using such rare events as an indicator for assessing the quality of maternity care in developed countries is highly debatable [37, 38]. As a result of improved perinatal mortality statistics [39], the case for launching a radically altered perinatal care system does not seem evident anymore particularly with limited evidence and lack of peer-reviewed literature informing this radical shift in the Dutch maternity system. Nevertheless, rare and significant events such as perinatal mortality can provide an important starting point for in-depth studies aimed at understanding key issues relating to the care system. Our study contributes to that understanding.

Implementation of a new system can only be successful if there is support for change among all professionals and clients concerned [7, 40], and all stakeholders are ‘market ready’ [41] (Bruijnzeels; personal communication). There have been calls from different stakeholders to reconsider introducing integrated maternity care [40, 42]. Furthermore, it should not be forgotten that horizontal integration (cross-sectorial collaboration within primary care) is also key to counteracting the fragmentation of services in the health system [27, 43, 44]. Health care systems with a strong emphasis on primary care are more likely to provide better population health, greater economy in the use of resources and better distribution in health throughout the populations [45]. Birth models that are ideologically and practically based on the midwifery-led care (humanistic/holistic) model of maternity care produce better outcomes for mothers and babies than technocratic practices based on a more medical model of maternity care [2, 46–50], but they are also fragile and in need of more attention and valuation [51]. The functions of primary midwifery care, such as first contact, comprehensiveness and coordination, and the person and population health-focused view could give primary care a central role in coordinating and integrating care [2, 27]. Many countries saw responsibility move from midwives to obstetricians over the 20^th^ century and in later years calls for more natural childbirth and more community based maternity services have contributed to a trend towards reintroducing or strengthening the roles of midwives [52]. Primary care midwives can be more aware of this added value they bring and advocate it more strongly in the collaboration with other care providers [40].

A limitation of our study might be that all but one of the interviewees were acquainted with the interviewers and that the researcher CW had a dual role as researcher and educator (but not examiner) with some interviewees. This ‘power-over’ relationship might have influenced the interviews; yet, it did not discourage the students from expressing positive as well as negative feelings and opinions and we do not believe the influence was negative or coercive in nature. The so-called investigator-triangulation (the collaboration of a qualitative reflexive interviewer (CW) with peer-interviewers (YC, MR)) brought together various ways of knowing (knowledge of the organisation of maternity care, the students midwives and academic researchers). The peer-interviewers improved the richness of qualitative data because they were able to establish deep rapport with participants, which enhances the process of sharing personal stories.

Another limitation might be that the focus group participants were familiar to each other. Their relationship may have caused participations to withhold certain experiences from their fellow students, and therefore from the focus group. On the other hand, it may have facilitated the discussion because they already knew each other. Our study did not include students from other midwifery academies.

The aim of our study was the empirical exploration of perceptions towards future maternity care models from the viewpoints of a sample of future midwives. However, trends for the future might mostly not be designed by the professionals-to-be who actually experience the changes in practices first hand, but imposed by legislative and governmental institutions or by highly placed iconic individuals [3, 5, 10]. Although we perceive the expectations of our target group to be valuable, one should thoughtfully interpret and use these expectations for future maternity care models. Expectations are not facts, and thus they might not come true. Nevertheless, it can be important to involve students’ views in the discussion, because student midwives are on the threshold of going to work as maternity care professionals. The findings of this qualitative study can provide the context for the planning and interpretation of a web-based survey among the whole population of final year student midwives in the Netherlands.

## Conclusions

Our qualitative study on student midwives’ perceptions towards future maternity care models indicate that students are open to change and that shifts in the organization of maternity care would be facilitated by inter-professional collaboration. Students believed in the importance of client-centred care and the physiologic, normalcy approach to pregnancy and birth, regardless of model of care. Students were also motivated to expand their scope of practice in order to enhance their role in providing intrapartum care. It can be important to involve students’ views in the discussion, because they are the future midwives and are entering into a changing maternity care system. A survey among the whole population of final year student midwives in the Netherlands is recommended.

## Abbreviations

AVAG: Midwifery Academy Amsterdam and Groningen
CPZ: College Perinatal Care
CTG: CardioTocoGraphy
EFPC: European Forum of Primary Care
HIRC: Healthcare Interdisciplinary Research Conference
KNOV: Royal Dutch Organization of Midwives
NIVEL: Netherlands institute for health services research
NVOG: Dutch Society of Obstetrics and Gynaecology
VAA: Midwifery Academy Amsterdam
VAG: Midwifery Academy Groningen
